# Prevalence of smokeless tobacco use in India and its association with various occupations: A LASI study

**DOI:** 10.3389/fpubh.2023.1005103

**Published:** 2023-02-27

**Authors:** Bhavna Bharati, Kirti Sundar Sahu, Sanghamitra Pati

**Affiliations:** ^1^KIIT School of Public Health, Bhubaneswar, India; ^2^School of Public Health Sciences, University of Waterloo, Waterloo, ON, Canada; ^3^Indian Council of Medical Research (ICMR)-Regional Medical Research Centre, Bhubaneswar, India

**Keywords:** smokeless tobacco, India, aging, occupation, tobacco consumption

## Abstract

**Background:**

More than two-thirds of deaths in developing countries are due to non-communicable diseases, and tobacco is a leading risk factor. There are numerous different socio-demographic factors that impact on the use of smokeless tobacco, of which occupation is one. The objectives of this study are to find out the overall prevalence of smokeless tobacco use (ever and current use), the pattern of association with various occupations and related variables (current and past workers), and the role of childhood adversity on initiation and use.

**Methods:**

This study used data from the Longitudinal Aging Study in India (LASI) wave 1, a nationally representative cross-sectional study collected in 2017–18. Current and previous users of smokeless tobacco are taken into consideration as the target population. For the data analysis, survey-weighted tools have been applied for descriptive statistics and multivariable logistic regression model. The weighted data analysis has been done using R studio with R version 4.

**Results and discussion:**

From the sample size of 65,561, 38% have used either smoking or smokeless tobacco. Among them, 40% use tobacco in smoke form, 51% use smokeless tobacco, and 9% take both. At the population level, 22.8 and 20.4% are previous and current users of smokeless tobacco, respectively. Type of occupation, type of employer, place of work, kind of business, and workload were found to be significantly associated with smokeless tobacco use. A deaddiction and tobacco quitting policy targeting rural male informal workers should be the focus of the Government.

## Introduction

Worldwide, consumption of smoked and smokeless tobacco (SLT) is a considerable threat to public health, leading to eight million deaths every year and being a health priority in many countries (1, 2). More than two-thirds of the deaths in developing countries are due to non-communicable diseases (NCDs), and consumption of tobacco is a leading preventable risk factor (1). Chewing tobacco, alcohol consumption, and smoking are positively associated with cognitive impairment in an aging population. Tobacco is one of the most readily, legally available, and easily accessible substances (3). Despite several global policy measures, the tobacco consumption trend is not reversing, and it is estimated that by 2030 ten million premature deaths will be due to tobacco use per year. Developing countries contribute to nearly 70% of tobacco consumption and death (4, 5). Globally, India ranks third in tobacco production and consumes around half of it (6). The Global Adult Tobacco Survey (GATS), (7) a combination of nationalized surveys using a standardized protocol in different countries, including India, monitors and tracks tobacco use worldwide (smoking and smokeless). The prevalence of tobacco consumption in all forms has increased in developing countries, including India (8, 9). According to GATS, nearly 30% of Indian adults used tobacco in any form for the year 2016–17 (10). About 20 million adults (21.4% overall, 29.6% men, and 12.8% women) regularly consume some type of SLT (11). The prevalence of SLT use (21.4%) is twice that of smoking (10.7%) (7). The use of SLT is prevalent not only among men, but also in other vulnerable groups, like teenagers, children, and women of reproductive age (12).

More than 40 types of SLT, such as pan, paan masala, khaini, sarda, mawa, gutka, mishri, and gudakhu, are used in chewing, snuffing, and applying to the teeth and gums (3, 13). The SLT products used substantially in India are khaini (tobacco-lime mixture), used by more than ten percent of the smoking population, gutka (a mixture of tobacco, lime, and areca nut), used by nearly seven percent, betel quid with tobacco, used by six percent, and mishri, gul, and gudakhu, used by nearly four percent for oral application (7). SLT is wrongly perceived as a safer form of tobacco than smoking which leads to more use, both in terms of initiation and persistence (13). Consumption of tobacco is not solely due to an individual's behavior, rather it is a complex process influenced by a variety of factors including social, environmental, and psychological. Evidence shows tobacco use, irrespective of its form, mainly starts in adolescence (12). At the individual level, the determinants include gender, wealth index, caste, parental use, peer use, impact of advertisement, and place of residence (13). A research study from India in 2016 shows education and occupation are two important critical predictors of use of SLT (14).

The LASI study was conducted in India as a part of the global study, with one of the components to understand tobacco use, which is an essential aspect for many policy-level changes and health care facilities (15). As evidence shows, age, gender, education, religion, wealth status, place of residence, marital status, and social category (including occupation) impacts use of SLT (4). Though occupation plays a pivotal role in initiation and continuation of SLT, there is limited evidence available from India; to bridge this evidence gap, this study aims to understand the impact of different types and places of occupation on the use of smokeless tobacco. As the LASI data included populations aged 45 and above, this study is limited to an older age group only. The objectives of this study are: (i) to find out the overall prevalence of smokeless tobacco use (both previous and current users), (ii) its variation with socio-demographic variables, and (iii) the pattern of association of smokeless tobacco use with various occupation and related variables including childhood adversities.

## Methodology

This is an exploratory study design using data from the first wave of the Longitudinal Aging Study in India (LASI), a nationally representative cross-sectional study (16). The data was collected from all the states and union territories across India in 2017–2018. A total number of 72,250 individuals participated in this study, from more than 61,000 households. For the next 25 years, LASI will be conducted biennially. It is India's first aging study where data was collected from individuals aged 45 and above and their spouses regardless of their age. The sampling and recruitment strategy details have been described elsewhere (16). Data are collected in broad categories from household and individual interviews, physical measurements, and biomarkers. The LASI study was approved by the Indian Council of Medical Research (ICMR) Ethics Committee, and written informed consent was obtained from participants.

For this study, variables from the demographic, health behavior, work retirement, and pension modules from the “*individual questionnaire”* were considered for analysis. From the demographic module, age, gender, caste, residence, education, religion, marital status, and wealth index are taken into consideration. For the health behavior module, the smoking section of the individual questionnaire is analyzed. We have considered both current and previous users of smokeless tobacco of any kind for the health behavior module. Participants who smoked tobacco or used any kind of smokeless tobacco were separated from those who had never used any tobacco product. From the population who used any kind of tobacco (smoke or smokeless), only the SLT users were the target population for further analysis. One question asked, “*what type of tobacco product have you used or consumed?,” leading to the question? Do you consume any smokeless tobacco products such as chewing tobacco, gutka, or pan masala?”* Their perception of childhood health status and their family financial status were explored in the childhood health module.

Type of occupation includes the broad area of occupation as well as the specific kind of job within that area (for example, a white-collar job would include managers as a specific role), place of work, industry, type of employer, and workplace characteristics (for example, whether the work requires a physical or mental workload).

The retirement and pension module discusses type of work: “*What is your occupation? Please specify?”* The level of physical effort and working conditions are asked about for the main job. The respondents graded their experience from “*all the time”* to “*almost never.”*

For this study, we have only included people aged 45 years and above. As this is a nationalized survey with multistage sampling, weight has been assigned for each respondent based on multiple variables. To address true representativeness, a weighted analysis has been done. Unit level data has been exported, cleaned, and analyzed using R studio with R-software version 4 with the “weights” package (17). All the analysis was performed between previous and current users of SLT to understand user and quitter behavior.

For multivariate analysis, occupations were regrouped from eleven categories to four unique groups based on the type of occupation to assess its association with the use of smokeless tobacco. Occupation category-1 includes “*Legislators, senior officials, and Managers,” “Professionals,” “Technicians and associate professionals,”* and “*Clerks.”* Category-2 includes “*Service workers and shop and markets sales workers,” “Skilled agricultural and fishery workers,” “Craft and related trade workers,”* and “*Plant and machine operators and assemblers*.” Category-3 *includes “Elementary Occupations,” “Workers not classified anywhere,”* and “*Others.” Category- 4 includes currently unemployed individuals*.

## Results

A total number of 72,250 individuals participated in this study. As LASI mainly focuses on data collection from 45+ age populations, six percent of the population comes under the 18 to 44 age group; we excluded that group from analysis, as they are not from the representative sample. Nearly 49% of the participants are within the 45 to 60 age group, and 45% are from the 60+ age group. The sample size for this study is 65,561 (45 years+). Among them, 24,777 (38%) have used either smoking or SLT. When analyzed for the type of tobacco product use, 39.8% use tobacco in smoke form, 51.3% use SLT, and 8.9% both SLT and smoke tobacco. We included the populations using SLT and both smoke and SLT for our analysis. At the population-level, 14,090 (22.8%) and 12,461 (20.4%) are previous users and current users of SLT respectively. In SLT, 88.6% of users continue using SLT, with a quit proportion of 11.4%.

Table 1 describes the demographic distribution of SLT users. Among current and previous SLT users, the proportion of 60+ age group, male, rural, and poor economic status are more than their respective counterparts. The share of the elderly population is more than the younger population. With respect to gender, males are consuming more SLT compared to females. Among the four categories of castes, schedule tribes (ST) population are the maximum consumers, with a share of 31.6% in previously used and 28.4% among current users of SLT. In contrast, other backward classes (OBC) and general categories are the least frequent users of SLT in both cases. Place of residence also influences the use of SLT, as urban residents consume less than their rural counterparts. Economic status determines SLT use, with poor and poorest respondents being the most common users. In terms of education, people with 10+ years of education are the lowest users of SLT compared to other categories of education, and there is a one percent difference between no-schooling and 5 to 9 years of schooling. There is a minimal difference in SLT consumption between currently married and widowed. Stratifying the users based on religion shows Hindus and Muslims consume more SLT than Christians and others.

**Table 1:** Demographic variables and their association with current and previous smokeless tobacco users.

**Features**	**Categories**	**Previous users**		**Current user**	
		***N***	**%**	***N***	**%**
Age	45–59 years	7,097	21.4	6465	19.6
	60+ years	6,993	24.3	5,996	21.3
Gender	Male	8,485	31.0	7,425	27.5
	Female	5,605	16.0	5,036	14.4
Caste	Scheduled caste	2,574	26.2	2,340	23.7
	Scheduled tribe	3,214	31.6	2,761	28.4
	Other backward class	5,030	20.8	4,508	18.5
	General	2,771	20.9	2,407	18.4
Residence	Rural	10,582	26.5	9,459	23.8
	Urban	3,508	14.9	3,002	13.0
Wealth index	Poorest	3,274	26.1	2,966	23.5
	Poorer	3,213	26.1	2,911	23.7
	Middle	2,822	22.8	2,504	20.3
	Richer	2,639	21.9	2,297	19.5
	Richest	2,142	16.5	1,783	14.2
Education	No school	6,810	23.3	6,143	21.2
	<5 years	2,080	28.2	1,838	25.2
	5 to 9 years	3,334	24.9	2,898	21.8
	10 + years	1,866	16.0	1,582	13.8
Marriage	Currently married	10,522	23.1	9,339	20.8
	Widow	3,169	22.5	2,758	19.7
	Other	398	18.6	363	16.5
Religion	Hindu	10,563	23.3	9,460	20.8
	Muslim	1,740	23.5	1,567	21.4
	Christian	1,332	16.4	1,057	12.3
	Others	455	16.7	377	14.6

The quitting pattern can be explored by comparing the difference between previous and current users of SLT. In the age group of 45–59 years, 2% of the population quit SLT, while in the 60+ population the share is 3%. It can be seen that the quitting habit increases with age. The quitting rate of schedule caste and schedule tribe is around 3% in caste, while other backward classes and general are 2%. There is no difference seen in the quitting pattern between rural and urban, with quitting percentage around 3%. The quitting pattern among the four education level shows that <5 years and 5 to 9 years of schooling have a 1% higher quitting rate than 10+ years and no schooling.

**Figure 1:**
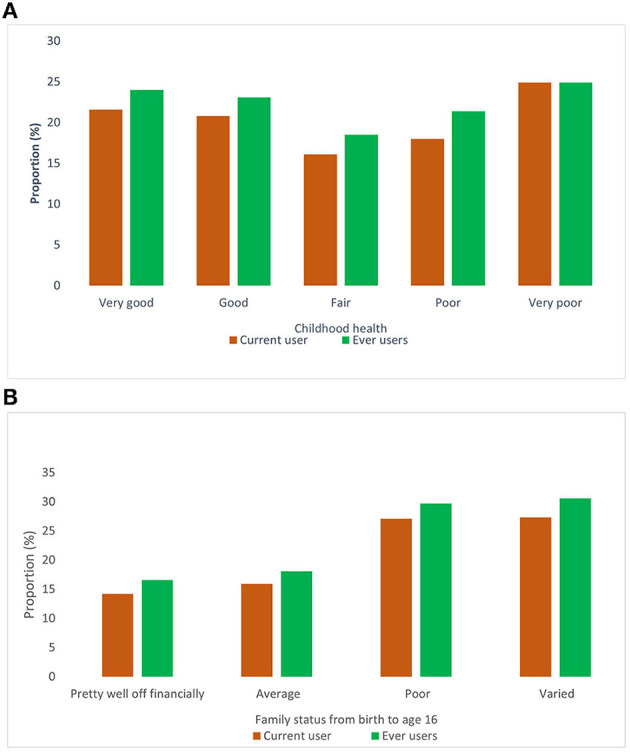
**(A, B)** Childhood characteristics (health and financial status) and its association with smokeless tobacco.

There may be childhood adversities that impact the initiation and consumption of smokeless tobacco. Two childhood features are considered, namely “childhood health” and “*family status in terms of finance, from birth to age 16*.” Family status is one of the most critical components that determines the use and the continuity of use of SLT. To find out the association between SLT use with childhood characteristics, further analysis is done and described below in Figure 1. As shown in the Figure 1A, in “*previous users,”* both the extreme categories of childhood health have a similar percentage of SLT use (24%). In terms of very good childhood health, the quitting pattern is 2.4%, while in contrast, the very poor category has no quitting pattern. Family status in terms of finance shows that poor and varied financial status is almost similar in terms of SLT use, with around 30 and 27% in previous and current use, respectively as seen in Figure 1B. It has been seen that there is no notable change in quitting pattern of SLT across the family status.

In addition to demography and family status, occupation and its correlates contribute to the use of SLT. Table 2 explains the association of type, place, kind of business, and type of employer with SLT. The analysis has been stratified between current and past workers for current users of SLT to understand the impact of occupational demands on the use of SLT. Table 2 focuses on occupational significance for SLT users in a broader aspect. We can see in types of occupation that skilled agricultural and fishery workers are the most common consumers of SLT in both current and past workers. Among past workers, plant and machine operators share the same percentage with agricultural and fishery workers (22%). Professionals are the lowest consumers of SLT in both current and past workers, with nearly 10% having consumed SLT. In legislators, senior officials, and managers for current workers, the share is nearly 9%, increasing to 17% for past workers. In the case of agricultural workers, the use of SLT is higher for current users and decreased by 7% points for past users.

**Table 2:** Impact of occupational on smokeless tobacco use for current users.

**Categories**	**Current workers**		**Past workers**	
	***N***	**%**	***N***	**%**
**Type of occupation**				
Legislators, senior officials, and Managers	25	8.97	42	17.2
Professionals	166	9.88	96	9.89
Technicians and associate professionals	88	13.3	62	12.6
Clerks	84	19.5	87	21.3
Service workers and shop and markets sales workers	648	21.3	209	18.2
Skilled agricultural and fishery workers	3,683	29.3	1,404	22.0
Craft and related trade workers	217	17.6	100	16.6
Plant and machine operators and assemblers	205	22.7	131	22.0
Elementary occupations	1,464	26.1	593	20.9
Workers not classified any where	829	26.2	391	1.4
Others	222	21.0	13	7.01
**Place of work**				
Own dwelling	898	21.7	388	15.1
Own farm or business	2,603	28.7	123	18.8
Employer's dwelling	249	25.3	647	19.0
Employer's workplace	791	21.5	111	19.6
Construction site	188	20.0	301	19.2
Place with fixed location	1,312	19.4	401	23.4
Place without fixed location	1,552	31.3	1,148	22.4
Other, please specify	43	19.6	14	17.6
**Kind of business**				
Agriculture, forestry, and fishing	4,542	28.2	1,861	21.9
Mining and quarrying	113	27.7	86	27.4
Manufacturing	299	22.9	141	17.8
Electricity, gas, steam, or air conditioning supply	49	23.7	29	16.4
Water supply: sewage, waste management	66	28.4	36	26.5
Construction	484	27.3	139	17.9
Wholesale and retail trade	617	22.8	167	19.6
Transportation and storage	208	25.2	60	15.7
Accommodation and food service activities	161	22.0	65	18.3
Information and communication	19	10.4	16	14.5
Financial and insurance activities	11	4.3	7	7.5
Real estate activities	26	24.5	12	27.5
Professional, scientific, and technical activities	56	14.2	37	8.5
Administrative and support service activities	81	13.4	55	17.3
Public administration and defense	55	10.9	58	14.7
Education	128	11.1	87	13.3
Human health and social work activities	76	17.8	32	16.5
Art, entertainment, and recreation	70	19.8	32	23.6
Other service activities	353	28.8	125	24.3
Activities of households as employers:	143	18.5	65	22.1
Activities of extraterritorial organizations and bodies	10	26.6	8	16.1
Other	69	18.5	12	16.5
**Type of employer**				
Government sector	464	16.2	384	15.7
Private sector/organization/entrepreneur	881	21.7	640	21.6
Cooperatives	27	23.5	25	23.0
NGO/trust	78	30.9	59	23.3
Individual household	363	23.2	481	23.7
Other, please specify	84	21.4	165	18.9

Regarding place of occupation, for current workers, people working without a fixed location have the highest share with 31.3%, followed by 28.5% for those that own a farm or business. In contrast, for past workers, people working with fixed locations outnumber other categories at 23.4%. Across all categories, SLT use for past workers is less than for current workers. When analyzed for kind of business for current workers, use of SLT is lower for financial and insurance activities, public administration and defense, education, information, and communication.

In contrast, previous or current use of SLT is high for agriculture, forestry, fishing, water supply, household employee, construction, and mining workers. For past workers, the pattern is nearly the same except for agricultural workers. In relation to the type of employer for current workers, people working in NGOs and trusts have the highest share (31%), whereas the share is lowest for people working in the government sector (16%). For past workers, the government sector has the lowest share, whereas all other categories have a similar share for SLT use.

**Figure 2:**
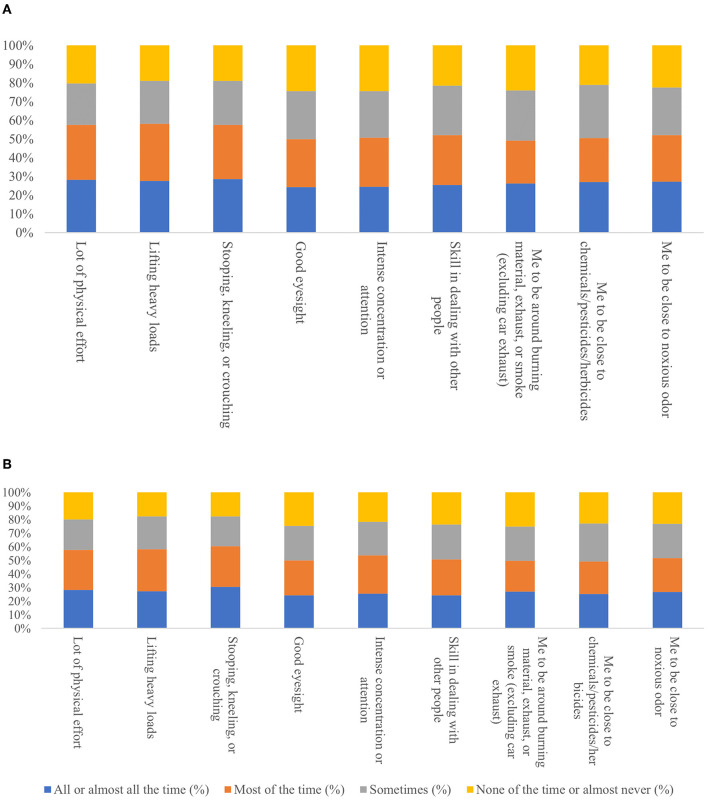
Physical effort and working condition required in **(A)** current & **(B)** past job for current smokeless tobacco users.

Figure 1 illustrates the impact of the different kinds of occupational characteristics in the work area. The demand for occupation plays a significant role in SLT use. When considering physical effort in current workers, those whose jobs involve constant physical effort have nearly the same use of SLT, and the same pattern is seen in past workers. As intensity of physical effort increases, so too does the use of SLT. Most of the time, those who lift heavy loads are the maximum consumers of SLT in both current and past workers, at 31 and 24%, respectively. Those working with chemicals are the leading consumers of SLT (31%) in current workers, and the same pattern is also seen for the past workers (23.6%). Figure 2 show no significant difference in the percentage of SLT use in current and past workers across the dedication of time of work required and requirement of eyesight.

The multivariate logistic regression analysis has been performed to understand the association of occupation on smokeless tobacco use after adjusting for age, gender, education, economic condition, caste, place of residence, zone to which the state belongs to, type of employer, childhood financial status, and childhood health. The result of the model, as shown in Table 3, shows that there is a statistically significant role of occupation on the use of SLT for current users even after adjusting for socio-demographic features and other variables. Adjusted and unadjusted Odds ratio for occupation has been mentioned in Table 3.

**Table 3:** Multivariate logistic regression analysis for current use of smokeless tobacco and occupation with other covariates.

	**Unadjusted odds ratio**	**Adjusted odds ratio**
**Occupation**		
Category-1	Ref	Ref
Category-2	2.6 (1.9–3.6)^***^	1.6 (1.2–2.0)^***^
Category-3	2.9 (2.1–4)^***^	1.5 (1.1–1.9)^***^
Category-4	1.4 (1–1.9)^∧^	1.1 (0.8–1.4)
**Age**		
45–59		Ref
60+		1.1 (1–1.2)^***^
**Gender**		
Female		Ref
Male		2.3 (2.2–2.5)^***^
**Income**		
Richest		Ref
Richer		1.2 (1.1–1.4)^***^
Middle		1.2 (1.1–1.3)^***^
Poorer		1.3 (1.1–1.5)^***^
Poorest		1.2 (1.1–1.4)^***^
**Education**		
10+ years		Ref
5 to 9 years		1.5 (1.4–1.7)^***^
<5 years		1.6 (1.4–1.8)^***^
No school		1.7 (1.5–1.9)^***^
**Residence**		
Urban		Ref
Rural		1.6 (1.5–1.8)^***^
**Caste**		
General		Ref
Schedule caste		1 (0.9–1.1)
Schedule tribe		1.2 (1–1.3)^*^
Other backward class		0.9 (0.8–0.9)^***^
**Employer**		
Non-government		Ref
Government		1 (0.9–1)
**Childhood health**		
Very good		Ref
Good		0.9 (0.9–1)
Poor		0.8 (0.6–0.9)^*^
**Childhood finance**		
Pretty well off		Ref
Average		1 (0.8–1.1)
Poor		1.6 (1.4–1.8)^***^

* *P* < 0.05;

*** *P* < 0.0001.

The odds ratio for the role of occupation was 2.6 for category-1, reduced to 1.4 after adjusting for other factors, which shows demographic variables have a strong impact on SLT use. The same can be seen for category 2/3.

## Discussion

The use of SLT has several direct and indirect effects on different dimensions of life. Occupation has a strong association with using SLT products. India, as an agriculture-dominated society, has a vast userbase of smokeless tobacco. With a sample size of 65,561 (45 years and above age group only), this study tried to explore the pattern of uses of SLT products in relation to occupation, demographic characteristics, and childhood adversities in India in 2018. Our study shows nearly 38% of the adult population (45 +) have used either smoking or SLT in the year 2018, which is almost 10% more compared to the Global Adult Tobacco Survey of India 2 (GATS-2), which was 28% in 2016–17. At the population level, 20.4% are current users of SLT, which is similar to the proportion of 21.4% by GATS-2 (11, 18).

The findings from our study of the demographic distribution of SLT users are on par with the findings from GATS-2, representing the robustness of SLT user's data across the nation. The typical characteristics of SLT users are aged 60+, male, rural, and with a poor wealth index. The elderly populations are more likely to be SLT users than the younger population by 2%, which is a matter of concern as elderly populations are more prone to other chronic health conditions, including cancer. With regards to gender, the share of males is almost double that of females in terms of SLT consumption in India. This inequality may be associated with employment status, as in India 71% of men are employed compared to 22% of women, as per the “periodic labor force survey” for 2017–18 by the Ministry of Statistics and Program Implementation (MOSPI) (19). Among the four categories of castes, schedule tribe are maximum consumers of SLT with 28.4%, followed by schedule caste with 24%, and a similar pattern can be observed in wealth index, with nearly 23% from both the poorer and poorest categories. In terms of place of residence, the rural population share the same percentage, respectively (23%). As we know, SLT use leads to both health and non-health complications; high use of SLT among socio-economically backward populations poses a threat to their self, family, and community. With this in mind, understanding the initiation and use of SLT as well as continuation has been explored as mentioned below.

Initiation of use of any tobacco product is assumed to be associated with childhood adversities, including health, finance, and socioeconomic characteristics. In our study, childhood health and family status are taken into consideration from birth to age 16. Our findings suggest that both parameters have a significant influence on the use of SLT. In later life, people with better health and financial status have higher quitting patterns than those with low economic status. Despite the understanding that the use of SLT can deteriorate existing poor health conditions, the negligible quitting pattern is a matter of concern for the healthcare system and society. Once imitated, SLT use continuation might be associated with occupation and its correlates and other associated factors.

When analyzed for the association between use of SLT and occupation, it is found that the type of occupation, place, type of work, kind of business, type of employer, and the occupational demands within the occupation has a crucial role, as evidenced in the past literature (14, 20, 21). Skilled agricultural and fishery workers have the highest share of SLT, while professionals, legislators, senior officials, and managers are the least frequent consumers of SLT. The reason behind this variation can be partly explained through social status, role model attitude, and peer environment. When all the members of a group are users of SLT, it is easier for a child to become used to tobacco as for them it would not seem unusual or harmful. Similarly, people with designated jobs are more conscious of their choice to use SLT in public, as opposed to people working in farming whose socioeconomic status is poor.

Along with the type of occupation, the work place also determines the use of SLT. For example, daily wage laborer has the highest share among SLT users compared to people working with “*fixed-locations.”* Similarly, “*kind of business”* also impacts the use of SLT, as our finding shows financial and insurance activities, public administration and defense, education, information, and communication business workers use limited tobacco products compared to agriculture, forestry, fishing, water supply, household employee, construction, and mining workers. Individuals working with insurance companies deal more with unnatural death like accidents and chronic diseases, and tobacco use is one of India's leading causes of mortality and morbidity. This could be the reason behind the lower use of tobacco by people working in insurance sectors. It may be due to their high health literacy and knowledge of the consequences of tobacco products (22).

People working in insurance sectors are more aware of the side effects of tobacco use as they are more health literate. As a developing economy, India has a high share of private companies, NGOs, and trusts as employers compared to a public entity (Govt.). The work environment varies from corporate-type setups to primary grassroots types of work like working in agricultural land or daily wage laboring. As our result shows, people working in NGOs and trusts have the highest share of SLT use; in contrast, the share is lowest for people working in the government sector. The crucial role of NGOs in India is to uplift the overall status of vulnerable populations in terms of health, economy, and education. All this requires intensive work in hard-to-reach areas, urban slums, and working with the homeless population, where the psychological status of the worker might not be good. SLT has also been associated with the type of physical and mental stress a person experiences during their work schedule. Those who endure much physical effort all or most of the time have high prevalent SLT use; for example, those who lift heavy loads, work with chemicals, or work with maximum eyesight requirements are the maximum consumers (23).

Multivariate logistic regression analysis shows that, compared to higher-level occupations, other groups were significantly at higher risk for the use of SLT. The role of gender seems to be extremely high even after adjusting with other variables, i.e., an odds ratio of 2.2 for males. Similarly, economic status, education, and place of residence have a significant role in determining the use of SLT. Interestingly, type of employer has no role, and the reason behind it might be due to the implementation of the anti-tobacco policy in government workplaces. Poor childhood financial status might have a significant role in initiating and continuing the use of SLT.

### Implication for workplace policy and healthy work environments

Central and state governments have implemented anti-tobacco policies to curb the use of smoking and SLT in the workplace since 2008. Our result shows occupation and its covariates have a strong association with the use of SLT, as evidenced in the previous literature. Informal workers without a fixed location have no tobacco policy in place, resulting in no difference in SLT intake. A deaddiction and quitting policy targeting rural male informal workers should be the focus of the Government.

The limitation of this study is the response related to occupation and its correlates and the use of SLT, which are self-reported and not validated by external sources. As the primary goal of LASI is to collect information from the geriatric/elderly population, the result cannot be generalized for the whole adult population. If using this result for policymaking, GATS result should be used to compare and complement the findings. Under-reporting is an important limitation when studying smoking and related behavioral factors. It has been proven that individuals are more likely to forget their own smoking habits, especially when they are former smokers (24).

## Conclusion

SLT use is a significant public health issue, and this study shows that a huge share of the population uses SLT in different form. Several Government policies are in place but are unable to completely control the use of tobacco. It is interesting to see the variation in SLT use in different types of occupation. Moreover, the relationship of the condition in the workplace also determines the use of SLT. Often, workers who lift heavy loads are the maximum consumers of SLT. There should be policy-level changes focusing specifically on the workplace, as the workplace can be a contributing factor to the initiation of SLT use.

## Data availability statement

The data analyzed in this study is subject to the following licenses/restrictions: LASI dataset is available upon request form the IIPS. Requests to access these datasets should be directed to datacenter@iipsindia.ac.in.

## Ethics statement

The studies involving human participants were reviewed and approved by Indian Council of Medical Research (ICMR) Ethics Committee, and written informed consent was obtained from participants. The patients/participants provided their written informed consent to participate in this study.

## Author contributions

BB, KS, and SP contributed to the conception and design of the study. BB organized the database and wrote the first draft of the manuscript. KS performed the statistical analysis and wrote the results sections of the manuscript. All authors contributed to manuscript revision, read, and approved the submitted version.
